# Differences in knee flexor and extensor force and kinematic variables in rural versus urban area female students in Romania

**DOI:** 10.3389/fphys.2024.1152119

**Published:** 2024-05-03

**Authors:** Ioana Mădălina Petre, Hadi Nobari, Mircea Boscoianu, Bogdan Pelin, Anca Ionescu, Pablo Prieto González, Rafael Oliveira, Bogdan Oancea

**Affiliations:** ^1^ Department of Industrial Engineering and Management, Transilvania University of Brasov, Brasov, Romania; ^2^ Department of Motric Performance, Transilvania University of Brasov, Brasov, Romania; ^3^ Faculty of Sport Sciences, University of Extremadura, Cáceres, Spain; ^4^ Health and Physical Education Department, Prince Sultan University, Riyadh, Saudi Arabia; ^5^ Sports Science School of Rio Maior–Polytechnic Institute of Santarém, Rio Maior, Portugal; ^6^ Research Center in Sport Sciences, Health Sciences and Human Development, Vila Real, Portugal; ^7^ Life Quality Research Centre, Rio Maior, Portugal

**Keywords:** load, power, velocity, strength training, lower body, low-intensity high-volume, impulse, program design

## Abstract

The study aimed to identify and explain the typical differences in low-intensity high-volume resistance training (LIHV-RT) performances for major muscle groups between rural versus urban young female students to establish the relevant set of quantitative and qualitative resistance training parameters. The study sample included 46 recreational active female students at the Transilvania University of Brașov, (mean ± SD age, 20 ± 1 year; body mass, 60 ± 3 kg; height, 160 ± 4 cm) grouped urban vs. rural. The study used modified resistance exercise machines for the hamstring- and quadricep-group muscles, equipped with a dynamometer and sensors for identifying developed forces and accelerations. A number of 368 tests were performed, representing two attempts for each subject, for knee flexion and knee extension exercises, with two different loads. For the performance analysis some variables were considered: the maximum number of repetition until failure, maximum force developed, maximum acceleration, the duration of the set and the mean time per repetition. The maximum number of repetition to failure shows a significant higher value for rural than urban in case of knee flexion (d = 0.98 [0.32, 1.54] for load 1(L1) and d = 0.65 [0.03, 1.21] for load 2(L2)) and in case of knee extension (d = 1.89 [1.11, 2.48] for L1 and d = 1.67 [0.92, 2.25] for L2). The total duration of the sets shows a significant higher value for rural than urban in case of knee flexion (d = 0.84 [0.19, 1.39] for L2) and in case of knee extension (d = 1.46 [0.74, 2.03] for L1 and d = 1.56 [0.98, 2.14] for L2). Additionally we found differences in the quality of the relevant repetitions execution and in the impulse developed during the LIHV- MNRF sets. The study’s main finding was that there are differences in LIHV-RT performances knee flexion and knee extension antagonistic exercises, between rural and urban female students. We concluded that the obtained results allow teachers to understand the optimal design of RT programs for the different groups of participants, in order to adapt their teaching techniques so that their final objectives are achieved, insisting on particular aspects of the theoretical or practical contents.

## 1 Introduction

Life in rural areas differs from urban areas considering transportation, mobility, stress, employment and pollution. Therefore, it is plausible that these differences may also imply distinct patterns of physical activity practice. According to the current studies, rural residents’ physical activity levels are similar to those living in urban areas, although the latter devote more time to active leisure (Robertson et al., 2018). Parks et al. analyzed the correlations of physical activity among US adults of varying income levels and areas of residence and the results. They confirmed that the status of perceived barriers, social support, and environmental characteristics is correlated to physical activity. Hoekman et al. investigated the intensity of sport participation in the Netherlands by comparing urban and rural areas. The results indicated higher rates of weekly sport participation in rural areas than in urban areas. The study contradicted the research made by Van Tuyckom that observed lower sporting activity levels for rural than for urban residents. Kellstedt et al. suggested that, along with other factors, youth sport participation plays an important role in children’s daily physical activity in rural communities.

Resistance training (RT) is well-established as a primary interventional strategy for increasing muscle strength and mass across populations (Varović et al., 2021), and it is an efficacious method of improving muscular strength and hypertrophy in adult females (Hangstrom et al., 2020). Programmed resistance exercises can be executed with different training intensities and volumes, according to the training objectives of each individual. In this regard, it is essential to remember that neuromuscular adaptations are directly influenced by the volume and intensity of effort (Schoenfeld et al., 2015; Schoenfeld et al., 2017), and those can be manipulated by altering sets, repetitions, load and concentric/eccentric acceleration.

In this regard, one training strategy is the maximum number of repetitions to failure (MNRF) at low intensities, with low loads. This implies using low-intensity high-volume resistance training (LIHV-RT), combining a high number of repetitions with reduced rest periods that consequently induce high levels of metabolic stress (Krzysztofik et al., 2019). This type of training has been considered an adequate stimulus to develop muscle hypertrophy (Schoenfeld, 2013).

Several studies have examined the effects of training to voluntary muscular failure on muscular strength and hypertrophy compared to non-failure (Fisher et al., 2016; Sampson and Groeller, 2016; Martorelli et al., 2017; Caroll et al., 2018; Nóbrega et al., 2018; Caroll et al., 2019; Lasevicius et al., 2019; Vieira et al., 2019; Lacerda et al., 2020; Karsten et al., 2021; Grgic et al., 2022; Simion, 2022). Some studies reported that training to muscle failure results in greater increases in muscular strength or hypertrophy (Caroll et al., 2019; Lasevicius et al., 2019). Other studies suggest that both training strategies can produce similar improvement (Nóbrega et al., 2018; Grgic et al., 2022) or that training to failure has a detrimental effect (Caroll et al., 2018; Caroll et al., 2019). Furthermore, Schoenfeld and Grgic. (2019) hypothesized that training for muscle failure is more important as workloads decrease, due to the delayed recruitment of larger motor units.

Lasevicius et al. used 30% and 80% 1RM loads to compare the effect of training to muscle failure vs. non-failure and verified that training to failure promoted greater increases in muscle size in individuals training with low loads. Mitchell et al. found that leg extension exercise performed at 30% 1RM until failure similarly increased quadriceps muscle volume compared to high-intensity exercise (80% 1RM) and was superior to a 30% 1RM non-failure condition. So, it can be concluded that 30% 1RM to failure is as good for hypertrophy as higher loads to failure.

We decided to utilise a machine-based knee flexor/extensor resistance exercise protocol because the variability of the results can be controlled and the measurements are accurate. Used with free weights exercises, the weight training machines yield improvements in strength levels (Prieto-González and Sedlacek, 2021). Antagonistic muscle group training is one of the main strategies for large muscular groups’ resistance training and reflect that once the agonist’s muscle is stimulated there is increased activation of motor units in the synergistic and antagonistic muscles (Rooney et al., 1994; Carregaro et al., 2011; Maia et al., 2014).

To date, no study has analyzed the differences in low-intensity resistance training performances for a major muscle group, between rural versus urban young female students, in a representative homogenous sample. Different studies focused on the RT performances of a homogenous group of young females (Myer and Wall, 2006; Hurley et al., 2018; Metcalf et al., 2019; Hagstrom et al., 2020), but no one has investigated a MNRF approach utilizing LIHV resistance exercise. This is why the present research is focused on comparative analyses for two relevant antagonistic exercises, knee flexion and knee extension.

Therefore, the study’s objective was to identify the differences in LIHV leg flexor and extensor resistance training performance between rural and urban young female students to determine the resistance training parameters. The study’s novelty is related to: 1) performance comparisons on two groups of female students for two single-joint RT exercises using the MNRF strategy adapted to LIHV; 2) identifying some connections between the main performance parameters and the quality of execution, based on the analysis of the physiognomy of the sets and repetitions. We hypothesized that there are differences between the rural and urban students related to their RT performances.

## 2 Methods

### 2.1 Participants

**FIGURE 1:**
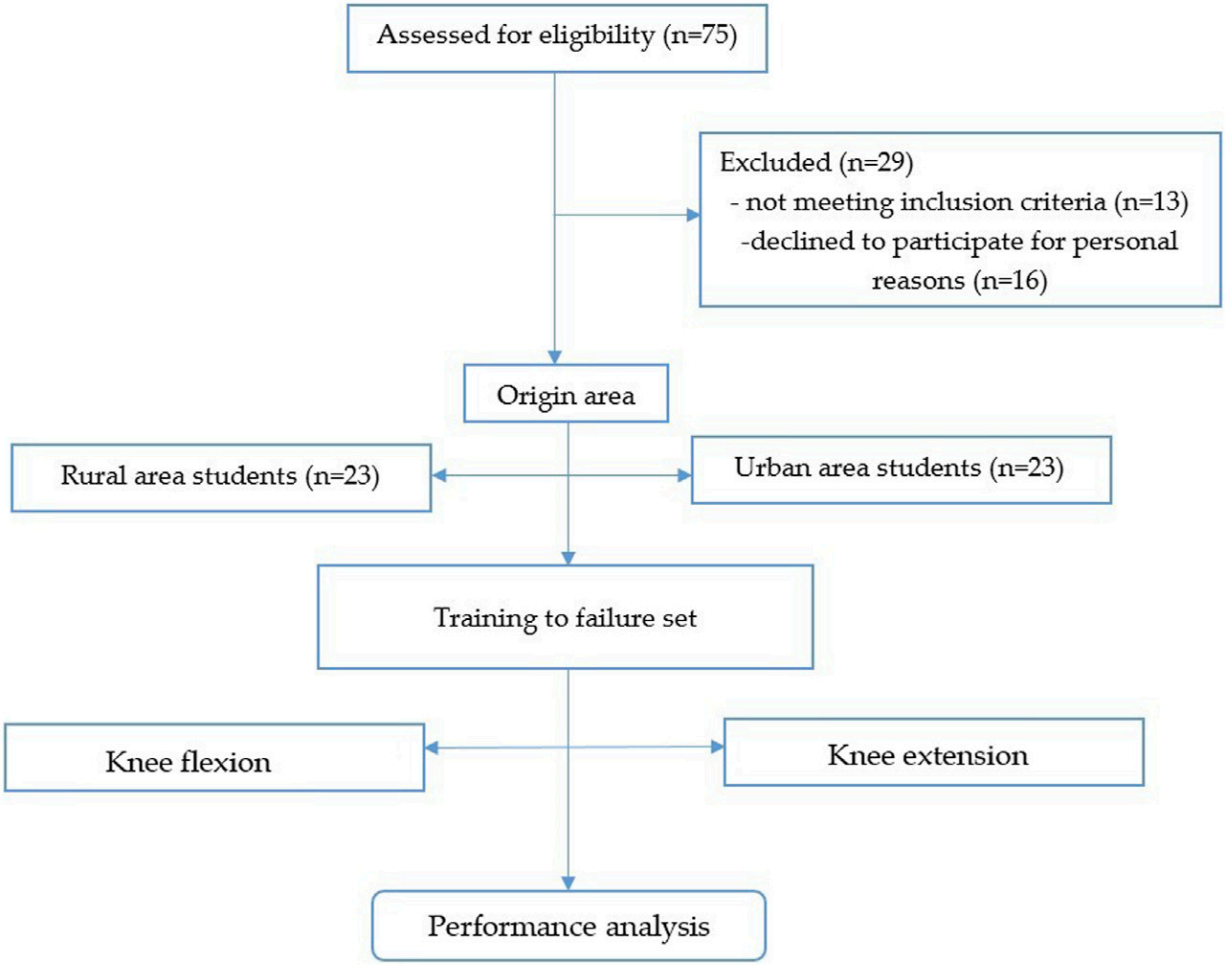
The flow chart of the experimental methodology.

The flow diagram of the research is presented in Figure 1.

A homogeneous sample of young female students at Transilvania University (Brașov) with relatively similar abilities was recruited. G-Power software (version 3.1.9.4, University of Düsseldorf, Düsseldorf, Germany) was used to calculate the sample size. T-test family sample power was calculated for *a priori* compute required sample size, considering an effect size of 0.4 for a recreationally trained population (Hurley et al., 2018) power (1–β) of 0.85 and an α level of 0.05. Thus, it was estimated that 40 participants would be required to conduct the present study. The total number of students initially recruited to participate in this study was 75. They all were active recreational individuals and, according to (McKay et al., 2022) belong to Tier 1 classification. The subjects were chosen considering the fact that the women are underrepresented in the research and also, considering the origin area. Subjects were identified as belonging to the rural and urban groups following the pre-test questionnaire. Subjects from the rural or urban groups have this quality only if they were born and lived all their lives in the respective environments. The percentage of the rural population in the Central area of Romania is close to 50% and this value is also preserved at the level of students of our university, falling within the national education strategy (ensuring access to higher education for all categories of beneficiaries).

The inclusion criteria were: 1) aged 18–24; 2) physical and sports practitioners at least once time a week (90 min) during the specific courses from the faculty curriculum; 3) no history of health problems or injuries in the last 3 months.

Some participants were excluded after the selection due to a loss of interest, personal issues or failure to meet the selection criteria. The final sample included 46 participants (20 ± 1.3 years; 60 ± 3.3 kg; 160 ± 3.7 cm), 23 from urban area and 23 from rural area, sedentary or recreational active females.

The subjects were informed of the research procedures and provided their informed consent. The local ethics committee of Transilvania University approved the experimental design. The fitness assessments were performed in the Department of Motor Performance of the Transilvania University of Brașov.

### 2.2 Experimental design

The study was conducted between April and June 2022. Strength parameters of the quadriceps and hamstring muscles were measured using one machine for knee flexion and one for knee extension purchased from Metal Fitness (https://www.metalfitness.ro). The chosen machines favor the correct execution of the exercises and, therefore, the optimal application of the MNRF strategy and can be loaded with standardized weights of 10 × 8 kg + 8 × 5 kg (maximum 120 kg).

The advantage of performing the exercises selected on the weight training machines is the uniformity of the executions, with the trajectories delimited by circular arcs. In contrast, when using free weight exercises, the weight training technique is not standardized, which may alter the results, especially in the case of MNRF strategy. Moreover, the selection criteria for the exercises included in the present study were: 1) antagonistic muscle groups; 2) simplicity; 3) ensuring that the exercises were performed with the correct form; 4) uniformity of execution.

For knee flexion, the subject was placed lying on the machine, with the legs under the resistance pads. The subject then bent her knees until reaching a 90-degree flexion. Next, the heels were slowly lowered to the starting position in the eccentric phase. The knee extension is executed sitting in a chair with knee extended to lift the pad and then return to the start position for the next repetition. In addition, 1–2 min (American College of Sports Medicine, 2009) of rest time between sets were given.

**FIGURE 2:**
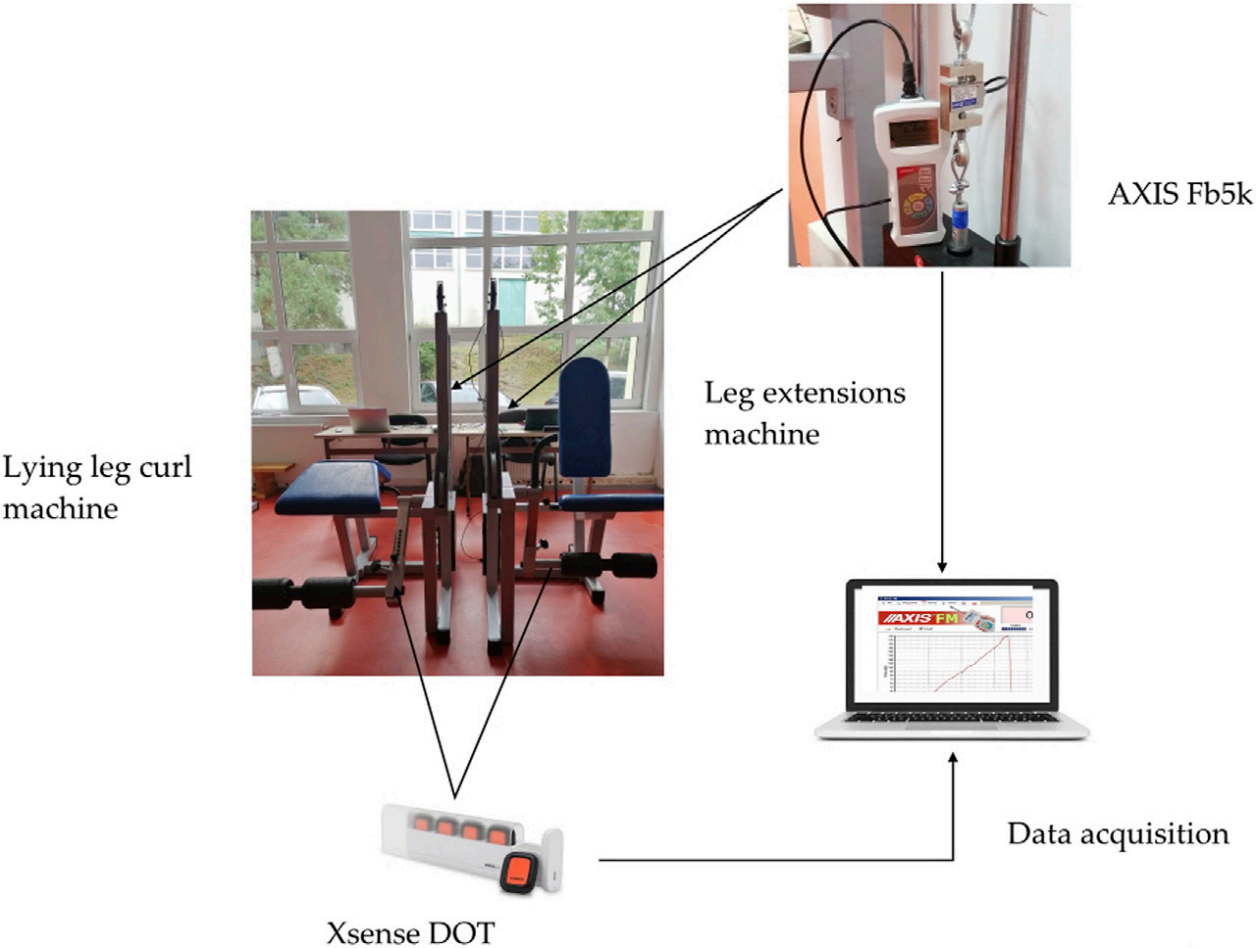
Experimental setup.

The experimental setup used for determining the forces and accelerations developed at muscle contraction consisted of several components, presented in Figure 2.

The dynamometer for determining the forces developed during the exercise was manufactured by AXIS FM (model Fb5k). The capacity of the dynamometer is 5000 N, and the accuracy ±0.1%. The values were obtained utilizing the Axis FM software, which allows reading the information on the display in real-time and recording measurements on the computer while the dynamometer is connected to the PC and the program is working. For the acceleration measurements, a Xsense DOT was used, which is a wearable sensor incorporating 3D accelerometer, gyroscope, and magnetometer. Next, the data was exported to one laptop. The sensor was attached to the machine, as can be seen in Figure 2 and the output rates have been set at 20 Hz.

The machine loads (L) used by the participants are 2 sets × 8 kg (L1) and 3 sets × 8 kg (L2). The machine loads were chosen considering the fact that the load required to increase maximal strength in untrained individuals is fairly low (American College of Sports Medicine, 2009). Light loads that can be lifted a maximum of 15–25 repetitions increased strength in moderately trained individuals (Rhea et al., 2003; American College of Sports Medicine, 2009). Previous studies demonstrated that when performing RT with lighter loads, a greater lifting volume (repetitions × load) is needed to reach volitional failure (Morton et al., 2016), or that when RT is performed to volitional failure, gains in muscle size or strength are independent of load (Stefanaki et al., 2019).

The protocol based on two attempts was designed to offer 48 h of rest for the muscle groups tested: Monday (L1) and Friday (L2) for knee flexion and Monday (L1) and Wednesday (L2) of the following week knee extension. The considered strategy consisted in performing the maximum number of repetitions in each set. In total, 368 measurements were performed in the MNRF strategy: 46 subjects x2 load machine x2 knee flexion attempts and 46 subjects x2 load machine x2 knee extension attempts. For each exercise and load, the best performance has been selected for the analysis.

For the performance analysis some variables were considered, such as: the maximum number of repetition (MNR) until failure, maximum force developed (F_max_), maximum acceleration (a_max_), the duration of the set (t_max_) and the mean time per repetition ( $\bar{t}$ ). These variables were collected through the data registered by the dynamometer and wearable sensor during the executions.

### 2.3 Statistical analysis

The statistical analysis was performed using the program SPSS Statistics for Windows (version 20.0, IBM Corp., Armonk, N.Y., United States). The results are presented as arithmetic mean (X), and standard deviation (SD). The assumptions of normality and homoscedasticity were verified with the Shapiro-Wilk and Levene’s tests. The T-test for independent samples was used to establish comparisons between groups, and the average differences between rural and residents (DX) were also calculated. The association between variables was performed using Pearson’s correlation test. Correlation values above 0.5 were considered strong, between 0.3 and 0.49 moderate, and below 0.29 poor (Cohen, 1988). Cohen’s (d) was used to estimate the effect size. The interpretation of the mentioned effect sizes was as follows: very small (d < 0.2), small (0.2 ≤ d < 0.5), medium (0.5 ≤ d < 0.8), and large (d ≥ 0.8) (Cohen, 1988). The significance level for all comparisons and correlations was *p* < 0.05.

## 3 Results

The results are presented considering the type of exercise and the machine load. Individual rankings were prepared for the two groups rural vs. urban and the parameters of interest such as forces and accelerations in their dynamic evolution during the repetitions were highlighted, as well as the durations of the repetitions and the MNRF set. Later, the impulse analysis was also integrated, as an element that brings additional clarifications for the qualitative aspects, particularly important in the case of this strategy.

The subjects’ anthropometric characteristics were measured before the tests (weight 60 ± 3.3 kg and height of 160 ± 3.7 cm). The rural subjects’ age was 19.8 ± 1.2, weight 59.1 ± 3.2 kg and height 161.1 ± 4.1 cm, while the urban group had 20.1 ± 1.4 years, 60.2 ± 3.3 kg and 159.4 ± 3.2 cm.

### 3.1 Knee flexion

First set consisted in knee flexion for hamstrings performances. The results are presented in Table 1. For the case of lighter load (L1), the largest difference between the two groups, in favor of the rural students, can be observed at the maximum duration of the set, 14.24 s. The MNR for rural students is 29.09 ± 12.39, *p* = 0.002; d = 0.98 [0.32, 1.54]; the local resistance is increased to the urban ones. The maximum duration of the set depends on the medium duration of a repetition and the number of repetitions performed by the various subjects. The medium speed of the repetition is difficult to be controlled in the case of MNRF strategy, but in this case the differences were reduced between the subjects or between the urban vs. rural groups.

**TABLE 1:** Statistical analysis of the developed parameters for knee flexion.

Load	Parameters	Groups	X	SD	DX	DDS	CI 95% Lower	CI 95% Upper	t	p	d	CI for d lower upper	
L1 [kg]	MNR [repetitions]	R	39.09	12.39	10.65	3.19	4.20	17.09	3.33	**0.01**	0.98	0.32	1.54
		U	28.43	9.02									
	F_max_ [kN]	R	0.16	0.05	−0.01	0.01	−0.04	0.01	−0.94	0.34	−0.29	−0.85	0.30
		U	0.17	0.05									
	a_max_[m/s^2^]	R	1.88	0.34	0.20	0.09	0.01	0.40	2.14	**0.03**	0.63	0.01	1.19
		U	1.67	0.30									
	t_max_[s]	R	89.09	25.34	14.24	7.50	−0.87	29.37	1.89	0.06	0.56	−0.05	1.12
		U	74.85	25.55									
	$\bar{t}$ [s]	R	2.58	0.92	−0.19	0.25	−0.70	0.32	−0.74	0.45	−0.23	−0.8	0.36
		U	2.78	0.80									
L2 [kg]	MNR [repetitions]	R	28.13	10.53	6.26	2.80	0.60	11.91	2.23	**0.03**	0.65	0.03	1.21
		U	21.87	8.36									
	F_max_ [kN]	R	0.24	0.08	−0.04	0.01	0.02	−0.09	−2.25	**0.02**	−0.69	−1.25	−0.06
		U	0.28	0.06									
	a_max_[m/s^2^]	R	2.03	0.41	0.18	0.13	−0.084	0.45	1.38	0.17	0.42	−0.18	0.98
		U	1.84	0.47									
	t_max_[s]	R	61.25	15.54	11.40	4.01	3.33	19.47	2.84	**0.01**	0.84	0.19	1.39
		U	49.85	11.27									
	$\bar{t}$ [s]	R	2.43	0.71	−0.07	0.19	−0.47	0.32	−0.37	0.71	−0.11	−0.68	0.47
		U	2.50	0.63									

Abbreviations: L, load; MNR, maximum number of repetition; F_max_, maximum force; a_max_, maximum acceleration; t_max_, maximum duration of the set; $\bar{t}$ , mean duration per repetition; R, group of rural areas students; U, group of urban areas students; X, average; SD, standard deviation; DX, mean difference; DDS, standard deviation of DX; CI, confidence interval; t, value of Student’s test; p, significant level of probability; d, effect size.

The significance level for all comparisons and correlations was *p* < 0.05. The values lower than 0.05 were bolded.

At the parameters analyzed, the results recorded by the rural students were higher than those of the urban students, except for the maximum force (F_max_) and medium time per repetition ( $\left.\bar{t}\right)$ .

An interesting aspect is that even if the number of repetitions executed by the rural students was higher, the forces developed by urban were greater to ensure rapid passage over the critical zone of repetition via compensatory acceleration and to enforce as many repetitions as possible.

The maximum duration of the set (t_max_) for rural was higher than the MNR. However, the average duration per repetition was lower for rural compared to urban. From a qualitative point of view, this shortening period of the repetition is explained by the economic skill of using the muscles in favor of the rural group, which tends to pass more quickly over the critical point of the repetitions. Comparative tests confirmed this aspect at the total impulse level throughout the set.

Regarding the results obtained with an additional 50% load (L2 = 1.5x L1), it can be observed that the most relevant differences in favor of rural students were registered at the MNR (28.13 ± 10.53, *p* ≤ 0.05, d = 0.65 [0.03, 1.21]), and the duration of the MNRF set t_max_ (61.25 ± 15.54, *p* ≤ 0.05, d = 0.84 [0.19, 1.39]). Similar to the previous load, the maximum forces developed by urban students are slightly higher (+28.6%), as well as the medium duration of the repetition (+3%). For L2, the shortening of the repetition duration by the rural group is much smaller than in the case of L1.

The analysis of the results of the statistical indicator Cohen’s for effect size for both samples showed a large effect size for t_max_ and a mean effect size for MNR, F_max_ and, respectively, a_max_; medium duration per repetition ( $\left.\bar{t}\right)$ had a small effect.

### 3.2 Knee extension

For the quadriceps exercise, the results are presented in Table 2.

**TABLE 2:** Statistical analysis of the developed parameters for knee extension.

Load	Parameters	Groups	X	SD	DX	DDS	CI 95% lower	CI 95% upper	t	p	d	CI for d lower upper	
L1 [kg]	MNR [repetitions]	R	53.83	13.15	20.56	3.18	14.15	26.97	6.46	**0.00**	1.89	1.11	2.48
		U	33.26	7.71									
	F_max_ [kN]	R	0.19	0.05	0.01	0.01	−0.01	0.04	1.16	0.25	0.35	−0.24	0.91
		U	0.17	0.02									
	a_max_[m/s^2^]	R	1.29	0.19	0.06	0.04	−0.03	0.15	1.27	0.21	0.37	−0.22	0.93
		U	1.23	0.11									
	t_max_[s]	R	107.1	37.87	49.51	9.94	29.46	69.55	4.97	**0.00**	1.46	0.74	2.03
		U	57.59	29.01									
	$\bar{t}$ [s]	R	2.12	0.75	0.36	0.21	−0.06	0.8	1.72	0.09	0.51	−0.11	1.07
		U	1.75	0.69									
L2 [kg]	MNR [repetitions]	R	36.22	10.57	13.17	2.31	8.50	17.84	5.68	**0.00**	1.67	0.92	2.25
		U	23.04	3.45									
	F_max_ [kN]	R	0.28	0.06	0.01	0.01	−0.01	0.04	0.95	0.34	0.3	−0.29	0.86
		U	0.27	0.03									
	a_max_[m/s^2^]	R	1.39	0.25	0.05	0.05	−0.06	0.17	0.86	0.39	0.25	−0.33	0.39
		U	1.33	0.13									
	t_max_[s]	R	70.98	23.48	33.18	6.24	20.60	45.76	5.31	**0.00**	1.56	0.83	2.14
		U	37.79	18.56									
	$\bar{t}$ [s]	R	2.11	0.59	0.47	0.17	0.11	0.83	2.65	**0.01**	0.78	0.14	1.34
		U	1.63	0.62									

Abbreviations: L, load; MNR, maximum number of repetition; F_max_, maximum force; a_max_, maximum acceleration; t_max_, maximum duration of the set; $\bar{t}$ , mean duration per repetition; R, group of rural areas students; U, group of urban areas students; X, average; SD, standard deviation; DX, mean difference; DDS, standard deviation of DX; CI, confidence interval; t, value of Student’s test; p, significant level of probability; d, effect size.

The significance level for all comparisons and correlations was *p* < 0.05. The values lower than 0.05 were bolded.

For the case of L1, some differences were observed between rural and urban students. MNR of rural students was 53.83 ± 13.15, with a mean difference (DX) of 20.56, *p* ≤ 0.01 and represented a large effect size (d = 1.89 [1.11, 2.48]). The duration of the sets was also much higher for the rural group (DX = 49.51, d = 1.46 [0.74, 2.03]). The maximum force developed by rural was 0.19 ± 0.05, d = 0.354 [−0.24, 0.91].

The results for the quadriceps performances at knee extension for L2 showed significant differences for the rural group: a mean difference of 13.17 at MNR (*p* ≤ 0.01, d = 1.67 [0.92, 2.25]) and a value of 70.98 ± 23.48 for the total duration of the set (*p* ≤ 0.01, d = 1.56 [0.83, 2.14])

There were significant differences in the mean duration of repetitions for this exercise and load (*p* ≤ 0.05, d = 0.78 [0.14, 1.34).

### 3.3 Comparative analyses for the physiognomy of the relevant repetitions—implications for the quality of entire set execution

#### 3.3.1 A qualitative analysis of the physiognomy of the relevant repetitions of the sets

Analyzing the physiognomy of the repetitions in MNRF strategy on the two loads provided quantitative benchmarks useful in understanding the differences between the two urban/rural groups. Starting from analyzing the physiognomy of the unique sets in maximum number of repetitions (MNR) strategy in the case of biceps as a small muscle group (Petre et al., 2022), the current paper targets the antagonistic muscles for the leg as the biggest muscular group.

A mediation of the obtained results was performed to analyze the differences between the initial and final repetition in the case of the two origin areas and the case of the two exercises (i.e., knee flexion for hamstrings and knee extension for quadriceps).

**FIGURE 3:**
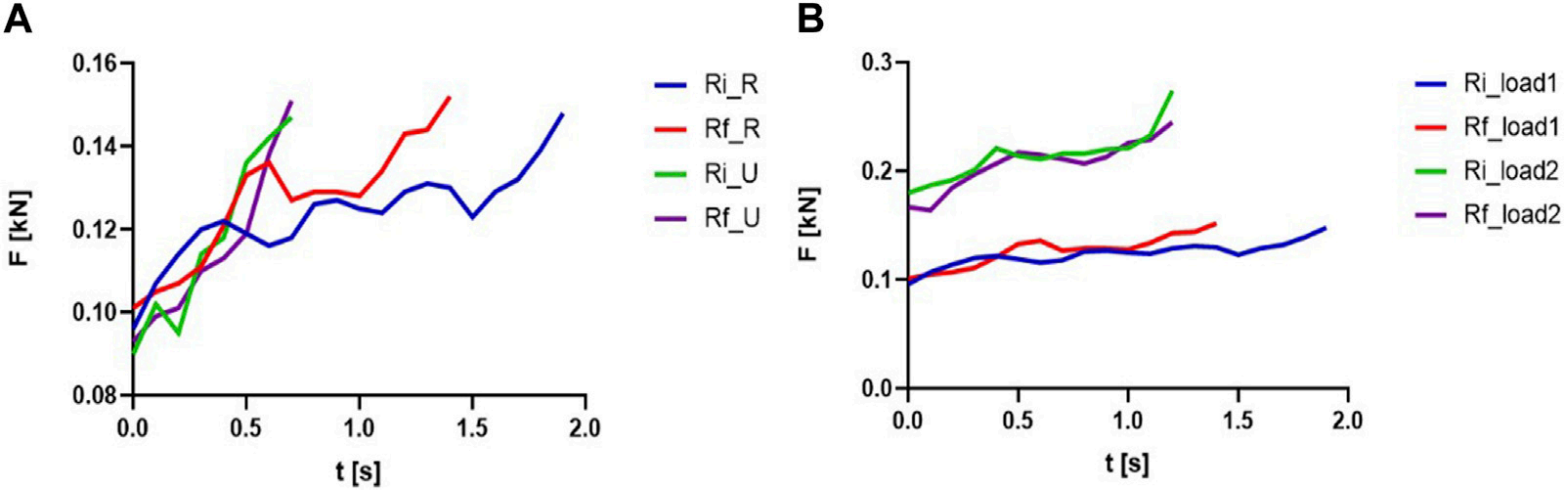
Superposition of the forces evolution at the initial (Ri) and final (Rf) repetitions: **(A)** knee flexion, L1, rural and urban students; **(B)** knee flexion, rural students, L1 and L2.

First, a superposition of initial and final repetition for the concentric phase for the knee flexion was made, for rural and urban students, for L1, as seen in Figure 3A.

As for L1, some differences between the first and last relevant repetition can be observed. In the case of rural students, it can be noticed that the time required to reach the maximum force value was about 30% longer at the beginning. In the case of urban students, there is no difference between the time taken to reach the maximum force value at the beginning or end of the execution.

Also, comparing the executions of the two groups (urban vs. rural), it can be observed that the time spent to reach the maximum force is double for the initial repetition in rural compared to the initial repetition executed by urban students. In contrast, the final repetition of the urban is about 70% shorter than that of rural.

The superposition of the initial and final repetition for the concentric phase for the rural students and exercises with different loads (i.e., L1 and L2) can be seen in Figure 3B. It is observed that the forces developed in the case of L2 are higher and the slopes are smoother than in the case of L1, but the duration of the concentric phases are similar. The initial repetition in the case of L1 is about 20% longer. These comparative elements offer an exciting image related to the quality of the execution but also the diversity of the execution modes that differ both for the selected exercises (i.e., knee flexion and knee extension), the proposed loads, and especially the rural vs. urban groups for which in the previous section were highlighted significant performance differences.

#### 3.3.2 A qualitative analysis based on the impulse developed during the LIHV- MNRF sets

By evaluating the impulse and using regressions for the concentric phase of the knee flexion and knee extension exercises, the results for various comparisons (load, exercises and urban/rural) were confirmed.

A comparison was made, between the impulse developed during the knee flexion exercise, during the concentric phase, with L1, using the medium values for the rural and urban groups of students. The mediation was performed considering the values for five relevant repetitions (i.e., the first R1, the last Rn, average Rm, and the two intermediate ones, Rim and Rmn) of the MNRF sets. In this way, we managed to capture the average attitude of the two analyzed groups in the context of focusing on the connection between performance and quality levels.

**FIGURE 4:**
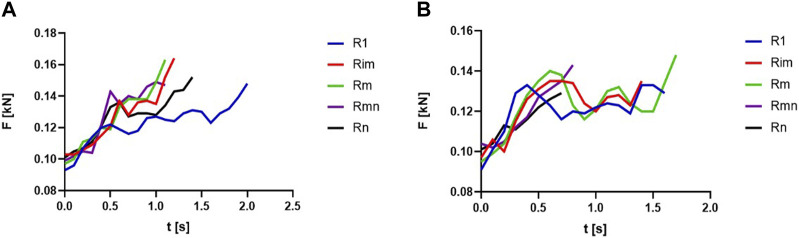
The evolution of the forces during concentric phases of repetitions: **(A)** rural, knee flexion, L1; **(B)** urban, knee flexion, L1.

Figure 4 presents the evolution of the forces during the concentric phases of the relevant repetitions in case of L1, for rural (a) and urban (b) students.

A polynomial regression represents the evolution of the forces in time for each relevant repetition, as seen in Table 3. Based on the polynomial regression developed, the impulse was calculated by integration, considering the time limits of reaching the maximal value of the force on the concentric phase. The medium value of the impulse, in case of rural students was 0.126 kN·s, and the total impulse, for the medium number of repetitions (MNR = 39) was 4.93 kN·s. In case of urban students, also, it can be seen the values of the impulse on each repetition and the medium and total impulse, 0.111 kN·s and 3.13 kN·s, respectively. MNR = 28 for the urban students.

**TABLE 3:** Polynomial regressions knee flexion_L1.

Area	Repetition	Polynomial regression	*R* ^2^	t $\in \left[0;n\right]$ [s]	Impulse [kN·s]
R	R1	$0.0825+0.0104∙t-0.0009∙t^{2}+2∙10^{-5}∙t^{3}$	0.92	*n* = 2	0.183
	Rim	$0.0934+0.0063∙t-0.0005∙t^{2}+3∙10^{-5}∙t^{3}$	0.92	*n* = 1.2	0.116
	Rm	$0.0859+0.0102∙t-0.001∙t^{2}+5∙10^{-5}∙t^{3}$	0.96	*n* = 1.1	0.1
	Rmn	$0.0937+0.002∙t+0.0011∙t^{2}-7∙10^{-5}∙t^{3}$	0.91	*n* = 1.1	0.104
	Rn	$0.0848+0.0129∙t-0.0014∙t^{2}+5∙10^{-5}∙t^{3}$	0.9	*n* = 1.4	0.13
				$\bar{I}$	0.126
U	R1	$0.0772+0.0172∙t-0.0019∙t^{2}+7∙10^{-5}∙t^{3}$	0.75	*n* = 1.6	0.143
	Rim	$0.076+0.0171∙t-0.0018∙t^{2}+6∙10^{-5}∙t^{3}$	0.79	*n* = 1.4	0.121
	Rm	$0.0683+0.0212∙t-0.0023∙t^{2}+7∙10^{-5}∙t^{3}$	0.77	*n* = 1.7	0.143
	Rmn	$0.1071-0.0061∙t+0.0023∙t^{2}-0.0001∙t^{3}$	0.99	*n* = 0.8	0.084
	Rn	$0.0961+0.0051∙t-0.0003∙t^{2}+2∙10^{-5}∙t^{3}$	0.96	*n* = 0.7	0.068
				$\bar{I}$	0.111

Abbreviations: R1, initial repetition; Rn, final repetition; Rm, mean repetition; Rim, first half intermediate repetition; Rmn, second half intermediate repetition; *R* ^2^, coefficient of determination; t, time; $\bar{I}$ , mean impulse.

**FIGURE 5:**
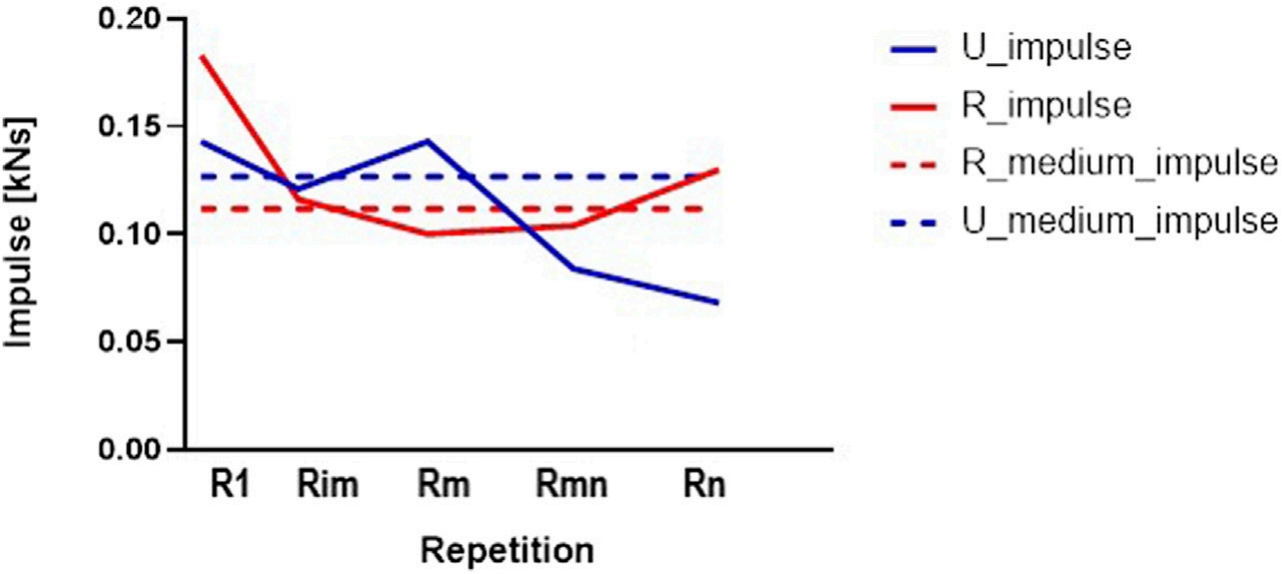
The longitudinal evolution of the impulse (concentric part of repetitions) for knee flexion—L1, rural and urban groups.

Figure 5 presents the longitudinal evolution of the impulse during the concentric phases of the five relevant repetitions (i.e., R1, Rim, Rm, Rmn, Rn) for knee flexion and knee extension, in a comparison between the rural and urban groups of students; dotted lines represent the medium impulse as the total impulse per number of repetitions. For urban students, in this case, a decrease is observed in the second half of the set in the conditions where the number of repetitions is lower than the case of rural students.

Another comparison was made between the impulse developed by the rural and urban students, in knee extension execution, with L2. Table 4 presents the polynomial regressions, the durations of the concentric phases at the relevant repetitions, and the impulses per repetition. Also, the total impulses were calculated by integration. For the rural students, the total impulse was 4.65 kN·s, considering a medium impulse of 0.129 kN·s and MNR = 36. In the case of urban students, the medium impulse was 0.148 kN·s, with a total impulse of 3.41 kN·s and a medium number of 23 repetitions.

**TABLE 4:** Polynomial regressions knee extension_L2.

Area	Repetition	Polynomial regression	*R* ^2^	t $\in \left[0;n\right]$ [s]	Impulse [kN·s]
R	R1	$0.0574+0.0638∙t-0.0077∙t^{2}+0.0003∙t^{3}$	0.91	*n* = 1.5	0.148
	Rim	$0.0808+0.0449∙t-0.0056∙t^{2}+0.0002∙t^{3}$	0.77	*n* = 1.6	0.179
	Rm	$0.0822+0.0411∙t-0.0053∙t^{2}+0.0002∙t^{3}$	0.91	*n* = 1.3	0.137
	Rmn	$0.0352+0.0698∙t-0.0083∙t^{2}+0.0003∙t^{3}$	0.92	*n* = 1.5	0.122
	Rn	$0.1407-0.0949∙t+0.0461∙t^{2}-0.0045∙t^{3}$	0.99	*n* = 0.5	0.06
				$\bar{I}$	0.129
U	R1	$0.0607+0.0595∙t-0.0085∙t^{2}+0.0004∙t^{3}$	0.96	*n* = 1.1	0.09
	Rim	$0.0753+0.0626∙t-0.0083∙t^{2}+0.0004∙t^{3}$	0.94	*n* = 1.2	0.13
	Rm	$0.039+0.0576∙t-0.0063∙t^{2}+0.0002∙t^{3}$	0.94	*n* = 1.7	0.139
	Rmn	$0.0675+0.0431∙t-0.0048∙t^{2}+0.0002∙t^{3}$	0.98	*n* = 1.5	0.144
	Rn	$0.0938+0.0333∙t-0.0033∙t^{2}+0.0001∙t^{3}$	0.93	*n* = 1.9	0.231
				$\bar{I}$	0.148

Abbreviations: R1, initial repetition; Rn, final repetition; Rm, mean repetition; Rim, first half intermediate repetition; Rmn, second half intermediate repetition; *R* ^2^, coefficient of determination; t, time; $\bar{I}$ , mean impulse.

**FIGURE 6:**
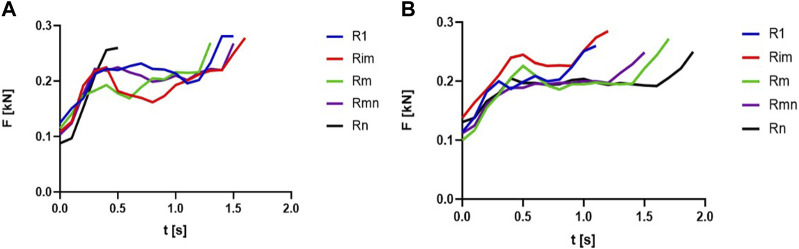
The evolution of the forces during concentric phases of repetitions: **(A)** rural, knee extension, L2; **(B)** urban, knee extension, L2.

Figure 6 presents the evolution of the forces, during the concentric phases of the relevant repetitions, in the case of the knee extension exercise, with a L2 load, for rural (a) and urban (b) students.

**FIGURE 7:**
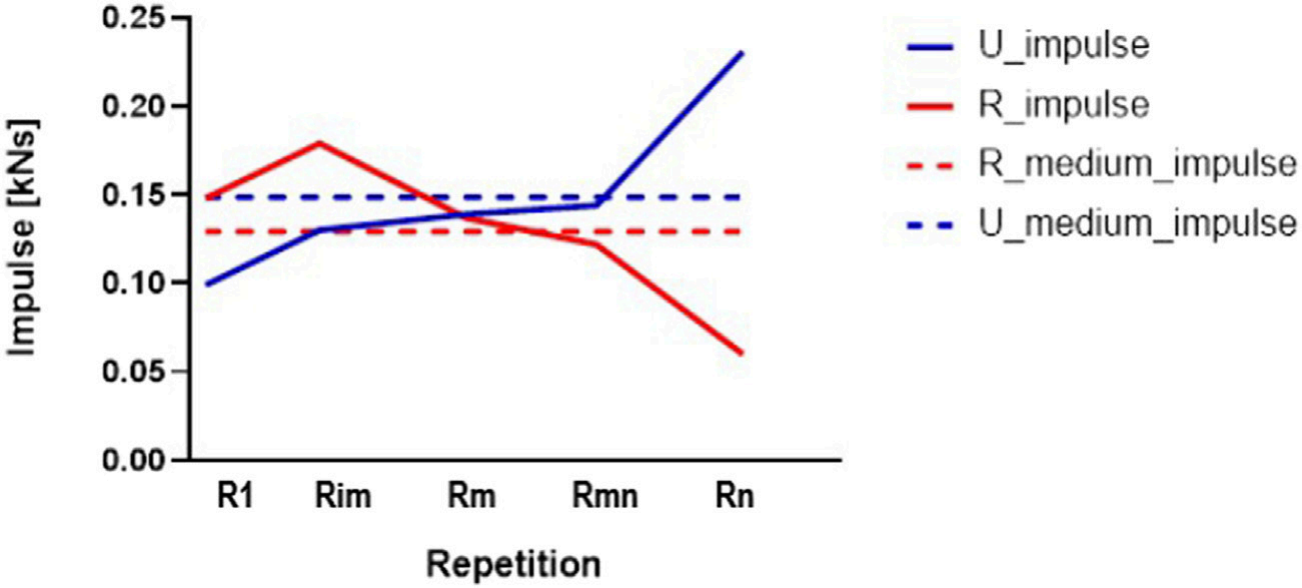
The longitudinal evolution of the impulse (concentric part of repetitions) for knee extension- L2, rural and urban groups.

In Figure 7 is represented the longitudinal evolution of the impulse during the concentric phases of the five relevant repetitions for knee extension with L2, for the same comparison between rural and urban. Although for 70%–75% of the repetitions the impulse was similar, for the last 25%–30% of the repetitions, the urban group showed an increase in the impulse whereas the rural group showed a reduction. The explanation is given by the superior effort made by the rural group to continue the execution of the knee flexion-L2 set by forcing the critical zone of the last repetitions performed. The comparative analysis showed that the urban group performed fewer repetitions than the rural group.

Table 5 presents the relevant elements of the performance comparisons. The results confirm the research purpose, showing significant differences in favor of rural compared to urban in an analysis based on two antagonistic machines- RT exercises for legs with two different loads.

**TABLE 5:** Differences centralizing table.

	Knee flexion				Knee extension			
	L1		L2		L1		L2	
	R	U	R	U	R	U	R	U
MNR	39.09	28.43	28.13	21.87	53.83	33.26	36.22	23.04
	37.4%		28.6%		61.8%		57.2%	
t_max_	89.09	74.85	61.25	49.85	107.1	57.59	70.98	37.79
	19%		22.8%		85.9%		87.8%	
$\bar{I}$	0.126	0.111					0.129	0.148
	13.5%						−12.8%	
$\bar{I_{t}}$	4.93	3.13					4.65	3.41
	57.5%						36.3%	

Abbreviations: L1, load 1; L2, load 2; R, group of rural areas students; U, group of urban areas students; MNR, maximum number of repetition; t_max_, maximum duration of the set; $\bar{I}$ , mean impulse; $\bar{I_{t}}$ , total impulse.

## 4 Discussion

The present study investigated the differences in low-intensity high volume resistance training performances for knee flexion and knee extension antagonistic exercises, between rural and urban female students. The results confirmed the research hypothesis which showed significant differences in favor of rural compared to urban, as seen in Table 5. The rural students had superior performances in terms of the maximum number of repetitions, duration of the sets and total impulse.

For knee flexion, the MNRF extra performance of rural was 37.4% (L1) and 28.43% (L2), but for knee extension, the differences were even higher (61.8% versus 57.2%). The duration of the LIHV-MNRF sets was also superior in favor of the rural group, 19%–22.8% for knee flexion and 85.9%–87.8% for knee extension.

In order to fulfill the objectives of the research, a judicious selection of the performed exercises was used, an application of the LIHV-RT strategy adapted for the targeted muscle groups, respectively an innovative methodology, which includes, in addition to the interpretation of quantitative data, a qualitative analysis based on impulse. Some studies have found that LIHV-RT provide a greater stimulus for hypertrophy and strength gains (Holm et al., 2008; Morton et al., 2016), but few studies have addressed the differences between rural and urban performances (Parks et al., 2003; Van Tuyckom, 2011; Hoekman et al., 2017; Robertson et al., 2018; Kellstedt et al., 2021).

Starting from the performance of a ranking for MNRF for the two exercises and the two loads, the dynamics at the level of effective forces, accelerations and impulses during individual repetitions and the entire unique set were determined.

When a submaximal contraction is sustained, motor units that were initially recruited will develop fatigue and produce less force or cease firing ultimately, necessitating the recruitment of additional motor units (Fallentin et al., 1993; Mitchell et al., 2012) to sustain force generation. The present study confirmed that LIHV RT provided an increase in the maximum number of repetitions, consistent with previous studies that demonstrated that when performing RT with lighter loads, a greater lifting volume (repetitions × load) is needed to reach volitional failure (Morton et al., 2016). Hackett et al. demonstrated a small to moderate the relationship between perceived exertion rate and momentary failure and a strong relationship between the estimated repetition to failure and short failure (Hackett et al., 2018).

For L2, the repetition duration by the rural group is much smaller than in the case of L1, and in a qualitative explanation, this is due to an approximation of the knee flexion execution modes executed by the two groups and a limited ability to use the muscles in an economical patter. Regarding the differences between the first and last relevant repetition, for the case of rural students, it can be noticed that the time required to reach the maximum force value was about 30% longer at the beginning of the execution. As the repetitions at lighter loads are repeated, the point of failure/fatigue ultimately necessitates near maximal motor unit recruitment to sustain muscle tension (Fuglevand et al., 1993). Considering the large number of repetitions executed by rural students, the reduction of the developed force is normal due to the onset of fatigue. In the case of urban students, there was no difference between the time taken to reach the maximum force value at the beginning or end of the execution.

The expectations regarding the superior performances of the rural group compared to the urban group were thus confirmed. For LIHV-RT, the MNRF strategy determined significant differences between the subjects in the two groups. Previous studies (Holm et al., 2008; Mitchell et al., 2012; Schoenfeld, 2013; Fisher et al., 2016; Morton et al., 2016; Sampson and Groeller, 2016; Martorelli et al., 2017; Carroll et al., 2018; Hackett et al., 2018; Nóbrega et al., 2018; Carroll et al., 2019; Krzysztofik et al., 2019; Lasevicius et al., 2019; Vieira et al., 2019; Lacerda et al., 2020; Karsten et al., 2021; Grgic et al., 2022; Simion, 2022) did not consider this type of urban vs. rural comparison.

In the present research, the subjects have similar age and anthropometric characteristics. Hence, the differences in LIHV-MNRF performance could be attributed to their belonging to the rural or urban area group. To understand the large differences in the relevant elements of performance, was also proposed an analysis based on the impulse, which is defined by the product of instantaneous force and time (Lake et al., 2014) and in the current study, it was computed by using the area under the net force-time curve during the concentric phase of each exercise. The total impulse and the impulse per relevant repetitions are important mechanical parameters that determine the magnitude and rate of motion of the object’s load with indications for the power (Knudson, 2009). The qualitative analysis of the impulses for the entire set based on the relevant repetitions allows comparisons regarding the execution mode of the sets for urban and rural under the conditions of impulse mediation and considering the differences at the level of the number of repetitions.

The present study contains some limitations that might be considered in future studies. First, the study included only female subjects aged between 18 and 24. Thus, the findings cannot necessarily be generalized to other categories of populations. The strength of the present study lies in the fact that a sample composed of recreationally active subjects from urban and rural areas was used. To the authors’ knowledge, this type of research is not currently available in the literature. Similarly, we collected a large amount of data to analyze the performance parameters and the quality of execution of the two relevant exercises of the -RT machine. In addition we should mention that the research carried out allows replication studies, the methodology being scalable and modular.

In summary, this study fills a knowledge gap regarding the comparative analysis of the LIHV- MNRF performance for the two interest groups, rural vs. urban subjects. In this study, the sets of data collected for LIHV-MNR (i.e., performance and quality of execution) showed interesting patterns, especially for knee extension. All these data allow an understanding of the optimal design of RT programs for different types of participants. Also, the obtained results gave a new vision of a new way of training students. It is a valuable indicator for the teacher to manage physical effort effectively and efficiently. Based on these results, future studies could extend the contributions to optimize strength training programs for people residing in different geographic areas. Moreover, it would be interesting to carry out a similar study with a different sample of men-subjects to compare the obtained differences in LIHV-MNRF performances.

Further studies are necessary to understand better the relationship between MNR in LIHV approach and 1RM and high or medium intensities (%RM) will confirm the differences rural vs. urban adding new explanations for understanding these significant differences recorded. Likewise, future work could be oriented on analyzing these differences after a 1–6-month period of RT workouts in a longitudinal approach.

## 5 Conclusion

The present research was focused on understanding the differences in LIHV-RT performances for knee flexion and knee extension antagonistic exercises, between rural and urban female students.

The most important difference between the two groups in favor of rural ones, was identified regarding the maximum number of repetitions until failure, the total duration of the sets, and the total impulse developed on the concentric phase of the repetitions for these unique MNRF sets. Considering the differences obtained between the two groups, teachers can adapt their teaching techniques so that their final objectives are achieved, insisting on particular aspects of the theoretical or practical contents that can be categorized as easy, without properly insisting on them.

## Data Availability

The raw data supporting the conclusion of this article will be made available by the authors, without undue reservation.
